# Supplementary material for: Modeling glioblastoma heterogeneity as a dynamic network of cell states
Source: Mol Syst Biol. 2021 Sep 16;17(9):e10105. doi: 10.15252/msb.202010105 (PMC8444284; doi:10.15252/msb.202010105)
Supplement: Supplementary file 6 — Source Data for Figure 5 [file MSB-17-e10105-s004.zip › Figure5A_sourcedata/GSEA_3071/hallmarks_stateV.GseaPreranked.1623742546329/HALLMARK_INFLAMMATORY_RESPONSE.html]

Details for gene set HALLMARK\_INFLAMMATORY\_RESPONSE[GSEA]

|  || Dataset | state43071 |
| Phenotype | NoPhenotypeAvailable |
| Upregulated in class | na\_neg |
| GeneSet | HALLMARK\_INFLAMMATORY\_RESPONSE |
| Enrichment Score (ES) | -0.51963496 |
| Normalized Enrichment Score (NES) | -2.5709438 |
| Nominal p-value | 0.0 |
| FDR q-value | 0.0 |
| FWER p-Value | 0.0 |
Table: GSEA Results Summary

  

Fig 1: Enrichment plot: HALLMARK\_INFLAMMATORY\_RESPONSE      
 Profile of the Running ES Score & Positions of GeneSet Members on the Rank Ordered List

  

| PROBE | GENE SYMBOL | GENE\_TITLE | RANK IN GENE LIST | RANK METRIC SCORE | RUNNING ES | CORE ENRICHMENT || 1 | PDPN |  |  | 65 | 1.493 | 0.0194 | No |
| 2 | SLC1A2 |  |  | 93 | 1.384 | 0.0438 | No |
| 3 | CD82 |  |  | 208 | 1.163 | 0.0468 | No |
| 4 | RGS16 |  |  | 348 | 1.018 | 0.0420 | No |
| 5 | CDKN1A |  |  | 363 | 0.993 | 0.0605 | No |
| 6 | ITGB8 |  |  | 564 | 0.822 | 0.0398 | No |
| 7 | SRI |  |  | 599 | 0.801 | 0.0504 | No |
| 8 | KCNJ2 |  |  | 1033 | 0.613 | -0.0192 | No |
| 9 | HAS2 |  |  | 1067 | 0.603 | -0.0126 | No |
| 10 | RAF1 |  |  | 1578 | 0.464 | -0.1001 | No |
| 11 | PDE4B |  |  | 1660 | 0.444 | -0.1061 | No |
| 12 | CALCRL |  |  | 1737 | 0.429 | -0.1114 | No |
| 13 | IL7R |  |  | 1761 | 0.425 | -0.1068 | No |
| 14 | CMKLR1 |  |  | 2245 | 0.357 | -0.1913 | No |
| 15 | PTPRE |  |  | 3014 | 0.267 | -0.3322 | No |
| 16 | CHST2 |  |  | 3126 | 0.258 | -0.3479 | No |
| 17 | SGMS2 |  |  | 3307 | -0.268 | -0.3765 | No |
| 18 | ITGB3 |  |  | 3508 | -0.301 | -0.4083 | No |
| 19 | TNFSF10 |  |  | 3651 | -0.323 | -0.4285 | No |
| 20 | TAPBP |  |  | 3712 | -0.333 | -0.4329 | No |
| 21 | TLR3 |  |  | 3825 | -0.354 | -0.4467 | No |
| 22 | ADRM1 |  |  | 3834 | -0.355 | -0.4407 | No |
| 23 | RIPK2 |  |  | 3867 | -0.362 | -0.4391 | No |
| 24 | PCDH7 |  |  | 3922 | -0.371 | -0.4415 | No |
| 25 | GNAI3 |  |  | 3965 | -0.381 | -0.4413 | No |
| 26 | ATP2B1 |  |  | 4126 | -0.416 | -0.4630 | No |
| 27 | HIF1A |  |  | 4130 | -0.417 | -0.4547 | No |
| 28 | NFKB1 |  |  | 4313 | -0.476 | -0.4793 | No |
| 29 | ABCA1 |  |  | 4333 | -0.483 | -0.4726 | No |
| 30 | IL15 |  |  | 4346 | -0.486 | -0.4646 | No |
| 31 | ADORA2B |  |  | 4535 | -0.556 | -0.4886 | No |
| 32 | ITGA5 |  |  | 4580 | -0.576 | -0.4847 | No |
| 33 | TNFAIP6 |  |  | 4764 | -0.688 | -0.5050 | Yes |
| 34 | HBEGF |  |  | 4822 | -0.734 | -0.5002 | Yes |
| 35 | CD55 |  |  | 4834 | -0.742 | -0.4865 | Yes |
| 36 | LIF |  |  | 4882 | -0.789 | -0.4786 | Yes |
| 37 | NAMPT |  |  | 4906 | -0.820 | -0.4655 | Yes |
| 38 | KLF6 |  |  | 4933 | -0.849 | -0.4524 | Yes |
| 39 | SPHK1 |  |  | 4997 | -0.928 | -0.4447 | Yes |
| 40 | MYC |  |  | 5020 | -0.972 | -0.4281 | Yes |
| 41 | EDN1 |  |  | 5038 | -1.014 | -0.4098 | Yes |
| 42 | LPAR1 |  |  | 5042 | -1.024 | -0.3885 | Yes |
| 43 | CD70 |  |  | 5050 | -1.031 | -0.3679 | Yes |
| 44 | MET |  |  | 5073 | -1.080 | -0.3491 | Yes |
| 45 | EMP3 |  |  | 5084 | -1.113 | -0.3273 | Yes |
| 46 | NMI |  |  | 5098 | -1.142 | -0.3054 | Yes |
| 47 | IRF1 |  |  | 5100 | -1.155 | -0.2810 | Yes |
| 48 | AXL |  |  | 5132 | -1.249 | -0.2603 | Yes |
| 49 | TIMP1 |  |  | 5134 | -1.251 | -0.2338 | Yes |
| 50 | MMP14 |  |  | 5146 | -1.297 | -0.2083 | Yes |
| 51 | SERPINE1 |  |  | 5161 | -1.377 | -0.1816 | Yes |
| 52 | PLAUR |  |  | 5185 | -1.479 | -0.1544 | Yes |
| 53 | TPBG |  |  | 5229 | -1.755 | -0.1252 | Yes |
| 54 | IL18 |  |  | 5237 | -1.816 | -0.0879 | Yes |
| 55 | F3 |  |  | 5242 | -1.927 | -0.0476 | Yes |
| 56 | INHBA |  |  | 5282 | -2.697 | 0.0025 | Yes |
Table: GSEA details [plain text format]

  

Fig 2: HALLMARK\_INFLAMMATORY\_RESPONSE: Random ES distribution      
 Gene set null distribution of ES for **HALLMARK\_INFLAMMATORY\_RESPONSE**
